# Chest radiography and computed tomography findings from a Brazilian patient with COVID-19 pneumonia

**DOI:** 10.1590/0037-8682-0134-2020

**Published:** 2020-04-03

**Authors:** Bruno Lima Moreira, Marcos Pama D’Almeida Brotto, Edson Marchiori

**Affiliations:** 1 BP - A Beneficência Portuguesa de São Paulo, Medicina Diagnóstica, São Paulo, SP, Brasil.; 2 Hospital Santa Catarina, Centro de Diagnóstico por Imagem, São Paulo, SP, Brasil.; 3 Universidade Federal do Rio de Janeiro, Departamento de Radiologia, Petrópolis, RJ, Brasil.

A 73-year-old man was admitted to the emergency department with a 4-day history of fever, chills, dry cough, and fatigue. He had arrived in São Paulo, Brazil, on the preceding day. His symptoms had begun when he was traveling in northern Italy with 12 friends, three of whom had been diagnosed with COVID-19. He reported having systemic arterial hypertension and type 2 diabetes mellitus. On examination, he had a temperature of 37.7°C, heart rate of 85 beats/min, respiratory rate of 15 breaths/min, blood pressure of 112/70 mmHg, and 94% oxygen saturation. His lungs were clear to auscultation. A leukogram was normal, and the patient’s C-reactive protein level was 4.78 mg/dL (normal levels below 1.0 mg/dL).

**FIGURE 1:**
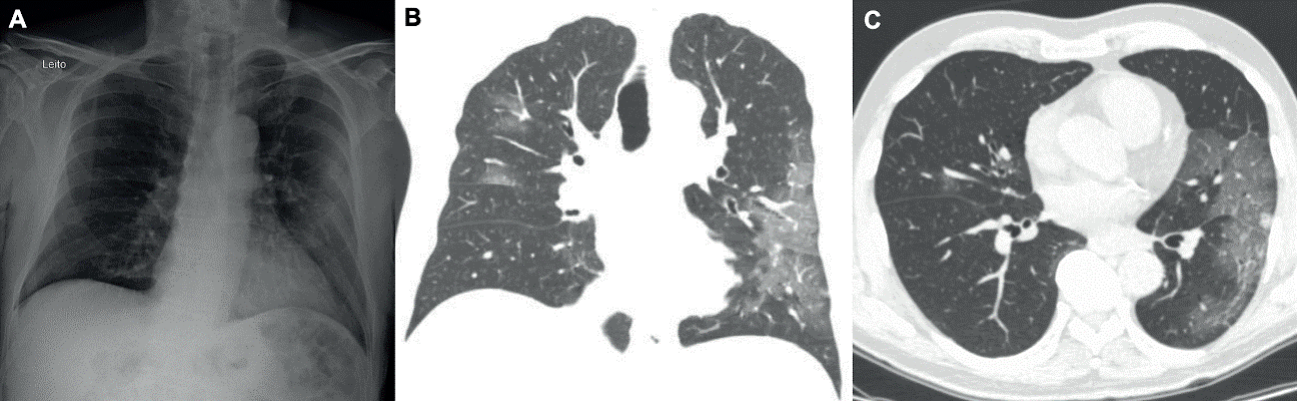
(A) A posteroanterior chest radiograph demonstrating ill-defined lung opacities, notably in the left lung. Chest CT images (lung window) in the coronal (B) and axial (C) planes show predominantly peripheral ground glass-opacities involving all pulmonary lobes, which are more exuberant in the left lung, where small foci of consolidation are also visible.

Chest radiography showed ill-defined lung opacities, notably in the periphery of the left lung. Chest computed tomography (CT) revealed predominantly peripheral ground glass-opacities involving all pulmonary lobes, which were more exuberant in the left lung, where small foci of consolidation were also seen (Figure 1). Real-time reverse-transcription polymerase chain reaction testing of a nasopharyngeal swab confirmed COVID-19 infection.

In December 2019, a novel viral pneumonia (subsequently named COVID-19 pneumonia) emerged in Wuhan, China^1^ ^-^ ^3^. It has spread worldwide, with an increasing number of deaths^1^ ^-^ ^2^. The main CT findings of COVID-19 pneumonia include predominantly peripheral ground-glass opacities, the crazy-paving pattern, and/or consolidation of the middle and lower lung regions, usually with bilateral and multilobar involvement^1^ ^-^ ^3^. Nonetheless, normal chest CT findings do not exclude this diagnosis^1^.
